# SARSCoV-2 antibody prevalence and titers in persons living with HIV cared for at a large tertiary reference center in Mexico City

**DOI:** 10.1186/s12985-023-02261-2

**Published:** 2023-12-15

**Authors:** Maribel Soto-Nava, Vanessa Dávila-Conn, Juan P. Venancio-Rocha, Pedro García-Esparza, Daniela Tapia-Trejo, Ramón Hernández-Juan, Eduardo Zarza-Sánchez, Akio Murakami-Ogasawara, Santiago Ávila-Ríos

**Affiliations:** Centre for Research in Infectious Diseases, National Institute of Respiratory Diseases, Calzada de Tlalpan 4502, Col. Sección XVI, 14080 Mexico City, Mexico

**Keywords:** SARS-CoV-2, COVID-19, Seroepidemiology, Vaccination, Antibody, HIV, Mexico City

## Abstract

**Objective:**

To assess SARS-CoV-2 antibody prevalence and titers in people living with HIV (PLWHIV) on antiretroviral treatment (ART) enrolled at a tertiary reference hospital in Mexico.

**Methods:**

Two plasma aliquots per person, used for HIV viral load follow-up between 01/2020 and 09/2021, were used to assess total anti-N and neutralizing SARS-CoV-2 antibodies. Sociodemographic, clinical, and SARS-CoV-2 exposure risk information were collected. The risk associated with SARS-CoV-2 exposure and associations with antibody titers were analyzed with logistic, Cox, and linear multivariable models.

**Results:**

803 PLWHIV participated; 233 had detectable SARS-CoV-2 antibodies (prevalent cases), and 132 seroconverted (incident cases). Overall, the adjusted prevalence was 46.45%, with an incidence rate of 3.78 cases/100 person-months. Factors associated with prevalent cases included lower age, location (western zone of Mexico City and the neighboring Mexico State), use of public transport, attendance at meetings without social distancing, and higher CD4 + T cell counts (*p* < 0.05; multivariable logistic model). BNT162b2 vaccination reduced incident cases (Cox adjusted HR = 0.4; *p* = 0.013). Notably, previously infected and vaccinated individuals showed maximization of neutralizing activity (*p* < 0.001). No associations between SARS-CoV-2 neutralization and HIV-related variables (CD4 + T cell counts, viral load, number of years in viral suppression, ART regimen) were found in multivariable analysis.

**Conclusions:**

SARS-CoV-2 infection was associated with community risk rather than HIV-associated variables in PLWH on ART and clinical follow-up. Antibody neutralization activity in vaccinated participants was maximized with previous SARS-CoV-2 infection.

**Supplementary Information:**

The online version contains supplementary material available at 10.1186/s12985-023-02261-2.

## Introduction

By the end of 2021, the COVID-19 pandemic, caused by SARS-CoV-2, had led to more than five million deaths globally [1], with 58% of people having received at least one dose of COVID-19 vaccine [2]. The emergence of new viral variants, the implementation of vaccination programs and the progressive return to daily life activities have modified COVID-19 epidemic patterns unevenly across countries. Four epidemic waves with peaks in July 2020, January 2021, August 2021, and January 2022 have occurred in Mexico [3]. Vaccination has had an important role in the distribution and clinical manifestations of COVID-19 cases, with fewer severe cases and lower mortality as the pandemic progressed. However, the prevalence of COVID-19 cases and the impact of the Mexican vaccination strategy in groups with higher vulnerability such as people living with HIV (PLWHIV) is unknown.

Mexico started its COVID-19 vaccination program in December 2020, using an age-stratified strategy that prioritized elderly people [4]. By October 2021, Mexico City, which concentrates nearly a quarter of the total national COVID-19 cases [3, 5], had one of the highest vaccine coverage worldwide, with 74% of adults having received at least one dose of any of the nationally approved vaccines [6].

HIV diagnosis and medical care for PLWHIV was significantly affected by the COVID-19 pandemic [7]. In Mexico City, the HIV epidemic is concentrated in key populations, predominantly men who have sex with men [8], who often arrive late to clinical care [9], possibly leading to higher susceptibility to SARS-CoV-2 infection and COVID-19 severity [10]. Nevertheless, assessing prevalence of SARS-CoV-2 infection and COVID-19 severity in PLWHIV with virological suppression and the associated impact of vaccination is also relevant, due to the long-term immune defects associated with chronic immune activation in treated HIV infection [11–13]. While some studies have reported similar, or even lower prevalence of SARS-CoV-2 infection in PLWHIV on virological control compared to the general population [10], asymptomatic infection rates may be underestimated. Moreover, the length and quality of the antibody response in PLWHIV after infection and/or vaccination remain incompletely understood [14].

Here, we present results of a retrospective longitudinal seroepidemiological study in PLWHIV on virological control, who attended an HIV-specialized tertiary reference center in Mexico City from January 2020 to September 2021.

## Methods

### Study design and setting

This is an observational, retrospective, cohort study among PLWHIV who receive clinical care at a large tertiary hospital, which also became the largest referral center for COVID-19 in Mexico City during the pandemic: The National Institute of Respiratory Diseases (INER). With the participants’ consent, plasma aliquots used for HIV viral load follow-up between January 2020 and September 2021 were used to assess SARS-CoV-2 antibody prevalence and titers. Two follow-up samples (the first and the last available) were selected for each participant within the study period.

### Study participants

We invited all PLWHIV cared for at the INER, who had previously provided their e-mail and/or telephone information and agreed to be contacted by the hospital staff (n = 1,126), to voluntarily enroll in the study. The participants were contacted by e-mail (n = 322), telephone (n = 98), text messages (n = 310), and by direct invitation at the moment of antiretroviral medication pick-up (n = 396).

Participants were asked to register in an electronic portal and answer a computer-assisted self-administered interview from June to September 2021. We collected sociodemographic data, information on community-associated risk of SARS-CoV-2 exposure, history of symptoms since the beginning of the pandemic, comorbidities, and ART interruptions. Laboratory data on HIV viral load, CD4 + T cell count, and antiretroviral treatment (ART) was obtained from the laboratory system. Unidentified data for analysis was stored in a local server, assuring confidentiality of participants’ information. Results from SARS-CoV-2 antibody tests were provided to each participant by personalized e-mails. The study was reviewed and approved by the Ethics Committee and the Ethics in Research Committee of the INER (Project Number: E01-21). All participants provided informed consent to participate in the study through the electronic portal designed for the protocol.

### SARS-CoV-2 serological tests

Cryopreserved, EDTA-anticoagulated plasma aliquotes collected for HIV viral load follow-up were used for SARS-CoV-2 antibody assays. We measured total anti-SARS-CoV-2 nucleocapsid (N) protein antibodies using an electrochemiluminescence-based commercial assay, as recommended by the manufacturer (Elecsys Anti-SARS-CoV-2, Roche, Basel, Switzerland), on a Cobas e411 instrument. Results were expressed as cut-off index (COI) values. A COI ≥ 1 was considered reactive. The reported test sensitivity is 99.5% and specificity 99.8% [15].

SARS-CoV-2 neutralizing antibodies were assessed using a commercial surrogate virus neutralization test (GenScript, Piscataway, NJ, USA), following the manufacturer’s recommendations. All tests were performed using 1:10 diluted plasma. Results were expressed as neutralization percentage. Percentages ≥ 30% were considered neutralizing. Neutralizing antibody tests were performed only with samples that resulted positive for total anti-N antibodies and for participants that reported having been previously vaccinated against COVID-19, independently of the total anti-N antibody test result.

### Statistical analyses

We defined three study groups among participants: prevalent cases (those who had a positive antibody test in their first sample), incident cases (those who had a positive test result in the second sample, after having a negative test result in the first sample) and non-cases (remained seronegative during the study period).

Seroprevalence was determined by calculating the percentage of individuals who tested positive for antibodies at any time during the study period, after adjusting for test sensitivity and specificity, as previously described [16]. The incidence rate was estimated by counting new positive cases in the second sample, excluding prevalent cases, and calculating person-time by adding up the time in days between samples 1 and 2 for participants with a negative result in the first sample. We used a Kaplan–Meier curve to report the time in days to the first positive sample for incident cases.

Sociodemographic characteristics, HIV follow-up laboratory data, presence of symptoms, and individual and community risk factors for acquiring SARS-CoV-2 infection were analyzed using medians and interquartile ranges or absolute counts and percentages, as appropriate. Univariate and multivariate logistic regression models were used to explore associations between these factors and SARS-CoV-2 infection for prevalent cases, while a Cox regression model was used for incident cases. Finally, neutralizing antibody titers were analyzed using a linear regression model. Multivariable adjustment included all variables showing significant bivariate associations, as well as relevant risk variables, age, and sex, which were included in the models a priori.

Analyses were performed using STATA v16, GraphPad Prism v6.0c, and R version 1·2·5019.

## Results

### Characteristics of the study participants

**Fig. 1:**
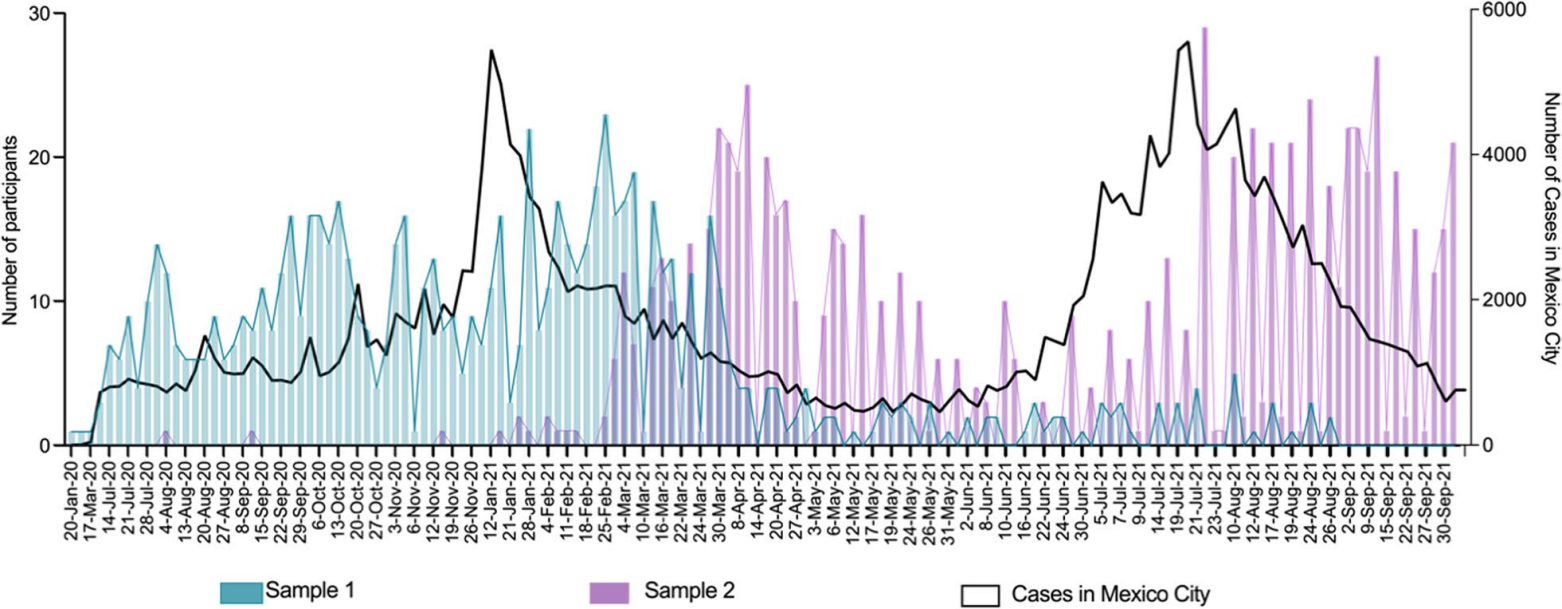
Sampling across the study period. Two plasma aliquots used for routine HIV viral load follow-up were selected for each participant from January 2020 to September 2021. The time frames of the first and second samples are shown in comparison with the official total number of COVID-19 cases reported in Mexico City during the study period [3]

**Fig. 2:**
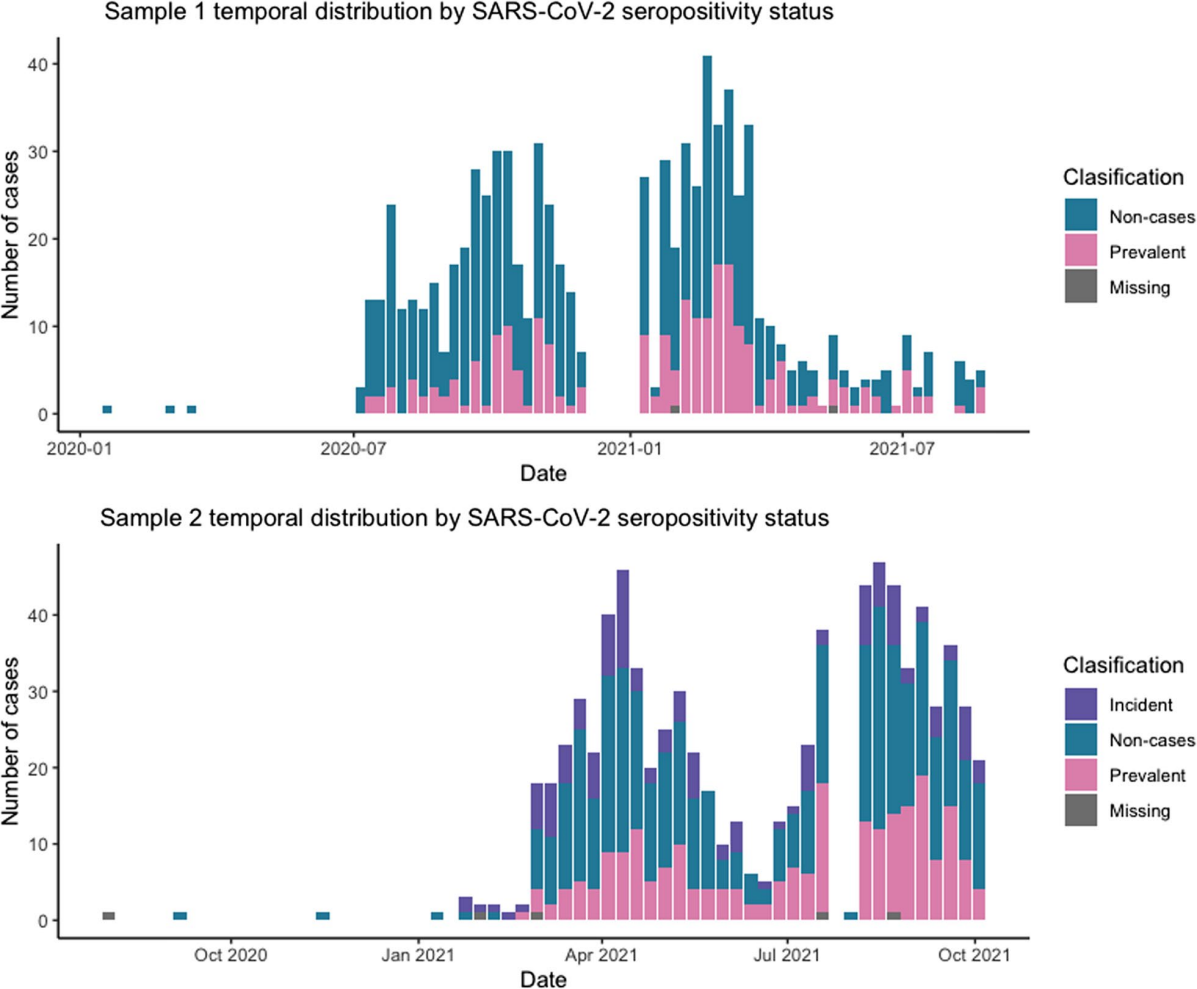
Prevalent and incident cases across the sampling period. Two plasma aliquots per person used for routine HIV viral load follow-up were selected for each participant from January 2020 to September 2021. The time frames of the first and second samples are shown. Bars are colored according to SARS-CoV-2 exposure group. Incident cases had a negative test in sample 1 and a positive test in sample 2

From a total of 1126 registered users, 803 were contacted and consented to participate in the study. The dates of sample collection coincided with the second and third waves of the COVID-19 epidemic in Mexico, with peaks reaching nearly 6000 daily new cases in Mexico City in January and July 2021 (Fig. 1). From the total of participants, 233 (29.0%) had detectable SARS-CoV-2 anti-N antibodies in their first sample (prevalent cases), and 132 out of the 570 still susceptible (23.2%) seroconverted between the times of donation of the first and second samples (incident cases). Prevalent, incident, and non-cases were evenly distributed across the study period (Fig. 2).

Participants were mostly cisgender men (82.2%), younger than 50 years old (70.6%), residing in Mexico City (68.8%) (Table 1). The median CD4 + T cell count at the time of the second sample donation was 435 cells/mm^3^, the median nadir CD4 + T cell count was 75 cells/mm^3^, and 93% had undetectable HIV viral load (< 40 copies/mL), with a median of 8 years of viral suppression (Table 2).

**Table 1:** General characteristics of PLWHIV participating in the study

		Total		Prevalent Cases		Incident Cases		Non-Cases		*p* value^b^
		n	%^a^	n	%^a^	n	%^a^	n	%^a^	
Age (years)	< 50	566	70.6	181	77.7	101	76.5	284	65	** < 0.001**
	≥ 50	236	29.4	52	22.3	31	23.5	153	35	
Sex at birth	Male	685	85.6	201	86.3	111	84.7	373	85.6	0.999
	Female	115	14.4	32	13.7	20	15.3	63	14.5	
Gender	Cisgender Men	659	82.2	188	80.7	107	81.1	364	83.3	0.527
	Cisgender Women	136	17	42	18	23	17.4	71	16.3	
	Transgender Women	3	0.4	1	0.4	1	0.8	1	0.2	
	Non-binary	2	0.2	0	0	1	0.8	1	0.2	
	Other	2	0.2	2	0.9	0	0	0	0	
State of residency	Mexico City	550	68.8	147	63.1	91	69.5	312	71.6	0.117
	State of Mexico	190	23.8	70	30	29	22.1	91	20.9	
	Other^c^	60	7.5	16	6.9	11	8.4	33	7.6	
Municipality	North Mexico City	92	11.5	24	10.3	13	9.9	55	12.7	0.163
	South Mexico City	171	21.5	39	16.8	27	20.6	105	24.2	
	East Mexico City	133	16.7	42	18.1	23	17.6	68	15.7	
	West Mexico City	151	18.9	41	17.7	28	21.4	82	18.9	
	State of Mexico	190	23.8	70	30.2	29	22.1	91	21	
	Other^d^	60	7.5	16	6.9	11	8.4	33	7.6	
Use of public transport	No use	352	43.9	87	37.3	63	47.7	202	46.2	**0.042**
	Subway	332	41.4	103	44.2	48	36.4	181	41.4	
	Taxi/Uber	24	3	5	2.2	4	3	15	3.4	
	Other^e^	94	11.7	38	16.3	17	12.9	39	8.9	
Travel by plane during the pandemic	Yes	141	17.6	46	19.7	23	17.4	72	16.5	0.353
	No	661	82.4	187	80.3	109	82.6	365	83.5	
Vaccinated against COVID-19	Yes	279	34.8	94	59.7	31	23.5	154	35.2	0.520
	No	523	65.2	139	40.3	101	76.5	283	64.8	
Type COVID-19 vaccine	BNT162b2 (Pfizer/BioNTech)	77	27.7	30	20.1	12	16.9	82	28.2	**0.001**
	AZD1222 (AstraZeneca)	128	46	84	56.4	40	56.3	134	46.1	
	Ad5-nCoV (Cansino)	13	4.7	6	4.03	7	9.9	13	4.5	
	Gam-COVID-Vac (Sputnik-V)	45	16.2	19	12.8	8	11.3	49	16.8	
	CoronaVac (Sinovac)	6	2.2	8	5.4	4	5.6	4	1.4	
	Ad26.COV2-S (Janssen)	5	1.8	0	0	0	0	7	2.4	
	Spikevax (Moderna)	4	1.4	2	1.3	0	0	2	0.7	

^a^Column percentages are shown

^b^Fisher exact test, two-sided p values are shown comparing all positive cases (prevalent cases + incident cases) vs non-cases. Statistically significant* P* values (≤ 0.05) are in bold

^c^Includes other states of Mexico

^d^Includes municipalities in other states

^e^Includes bus rapid transit (metrobus), bus, minibus and trolleybus

**Table 2:** HIV infection-related laboratory values of the study participants

	n	Prevalent cases		Incident cases		Non-cases		
		Median	(IQR)	Median	(IQR)	Median	(IQR)	*p* value^b^
Viral load (copies/mL)^c,d^	803	59	(44–65)	78	(49–133)	54	(47–107)	0.872
CD4 count (cells/mm^3^)^c^	801	430	(304–572)	437	(316–596)	438	(305–596)	0.664
CD4 percentage^c^	801	24	(18–30)	24	(17–31)	24	(18–30)	0.614
CD4:CD8 ratio^c^	801	0.7	(0.4–1.0)	0.7	(0.4–1.0)	0.7	(0.5–1.0)	0.989
CD4 nadir (cells/mm^3^)	801	79	(26–191)	95	(28–287)	69	(20–255)	0.416
Time on viral suppression (years)	803	7	(5–11)	7	(4–12)	9	(5–12)	0.107

*IQR* interquartile range

^b^Mann–Whitney U test, two-sided p values are shown comparing each group against the rest of the participants

^c^Results from sample 2

^d^93% of participants had undetectable viral load (< 40 copies/mL); medians and IQR of only participants with detectable viral load are shown

Most participants continued ART without interruption during the study period (98.1%), were mainly on bictegravir/tenofovir alafenamide/emtricitabine (75.3%), and 27.1% had comorbidities (Additional file 1). Regarding risks associated with SARS-CoV-2 infection, 26.3% were cigarette smokers, 40.6% reported attending meetings without social distancing, 28.6% reported COVID-19 symptoms requiring medical care (Additional file 1), 17% travelled by plane during the pandemic, 56.1% used at least one type of public transport, and 34.8% reported having been vaccinated against COVID-19 (Table 1). The most prevalent symptoms in persons with confirmed COVID-19 at any point of the study period (both for prevalent and incident cases) were fatigue, myalgia, and headache. As expected, anosmia, dysgeusia, dyspnea, and thoracic pain were highly specific of persons with COVID-19 compared to non-cases (Additional file 2).

### Overall prevalence estimation

Considering all the participants with a positive SARS-CoV-2 total anti-N antibody serological test result at any point during the study period (365/803), and correcting for test specificity and sensitivity, the adjusted prevalence in PLWHIV participating in the study was 45.57%. As expected, comparing the overall prevalence by epidemiological wave, we observed an increasing trend, with 16.1% in the first wave, 35.5% in the second and 46.6 in the third (Table 3).

**Table 3:** Proportion of positive total anti-N antibody tests by epidemiological wave

Wave	Sample 1 (n = 764)		Sample 2 (n = 645)		Total	
	n	%	n	%	n	%
First	29/178	16.3	0/2	0	29/180	16.1
Second	173/538	32.2	92/207	44.4	265/745	35.5
Third	19/48	39.6	207/436	47.4	226/484	46.6

For this analysis, samples obtained out of the timeframes of the epidemiological waves (n = 34 for sample 1 and n = 155 for sample 2) were excluded

^a^First wave: 2020/02/16 to 2020/09/26

^b^Second wave: 2020/09/27 to 2021/04/17

^c^Third wave: 2021/06/06 to 2021/09/23

### SARS-CoV-2 infection prevalence and associated risks of having a positive serological test at baseline

Using a multivariable logistic model, we assessed risk associated with having a positive SARS-CoV-2 serological test at baseline (Table 4). After adjustment for confounding variables, lower age (aOR = 0.97, 95% CI 0.95–0.99; *p* = 0.009), as well as living in the western zone of Mexico City (aOR = 1.8, 1.0–3.3; *p* = 0.04) and in the neighboring State of Mexico (aOR = 2.1, 1.2–3.5; *p* = 0.007) (compared to the south of Mexico City), were associated with higher odds of having a positive serological test at baseline. Persons using bus rapid transit, bus, minibus, or trolleybus, compared to persons not using public transport had higher odds of being prevalent cases (aOR = 2.1, 1.2–3.7; *p* = 0.012). Also, persons reporting attending meetings without social distancing had higher odds of a baseline positive test (aOR = 1.7, 1.2–2.5; *p* = 0.005). Drug use (excluding alcohol, tobacco, and intravenous drugs) showed a strong protective effect against being a prevalent case (aOR = 0.3, 0.1–0.5; *p* = 0.001).

**Table 4:** Logistic regression analysis on associated risks of having a positive serological test at baseline^a^

		n	(%)^b^	OR		95% CI		*p* value	aOR		95% CI		*p* value
Age (years)		N/A		0.98	0.96	–	1.0	0.012	0.97	0.95	–	0.99	**0.009**
*Municipality*	South Mexico City	39/171	26.4			Ref					Ref		
	North Mexico City	24/91	26.4	1.2	0.7	–	2.2	0.561	1.2	0.6	–	2.4	0.566
	East Mexico City	42/133	31.6	1.7	1.0	–	2.8	0.061	1.7	0.9	–	3.0	0.092
	West Mexico City	41/148	27.7	1.4	0.8	–	2.4	0.213	1.8	1.0	–	3.3	**0.040**
	State of Mexico	70/190	36.8	2.1	1.3	–	3.4	**0.003**	2.1	1.2	–	3.5	**0.007**
	Other^c^	16/60	26.7	1.3	0.6	–	2.6	0.456	1.1	0.5	–	2.3	0.887
*Comorbidities*^*d*^	No	167/581	28.7			Ref					Ref		
	Yes	66/217	30.4	1.0	0.7	–	1.5	0.867	1.2	0.8	–	1.8	0.424
	*Type of COVID-19 vaccine*												
	Non-vaccinated	81/277	29.2			Ref					Ref		
	BNT162b2 (Pfizer/BioNTech)	30/14	24.2	0.6	0.4	–	1.0	0.060	0.9	0.5	–	1.7	0.742
	AZD1222 (AstraZeneca)	84/256	32.8	1.1	0.7	–	1.6	0.710	1.5	0.9	–	2.3	0.095
	Ad5-nCoV (Cansino)	6/26	23.1	0.8	0.3	–	2.1	0.629	1.1	0.4	–	3.2	0.864
	Gam-COVID-Vac (Sputnik-V)	19/75	25.3	0.7	0.4	–	1.2	0.189	1.0	0.5	–	1.9	0.919
	CoronaVac (Sinovac)	8/16	50.0	3.4	1.0	–	11.6	0.052	26	2.9	–	227.7	**0.004**
*Meetings without social distancing*	No	132/474	27.9			Ref					Ref		
	Yes	101/324	31.2	1.2	0.9	–	1.7	0.254	1.7	1.2	–	2.5	**0.005**
	*Use of public transport*												
	No	87/351	24.8			Ref					Ref		
	Subway	103/329	31.3	1.3	0.9	–	1.9	0.103	1.1	0.7	–	1.6	0.659
	Taxi/Uber	4/24	20.8	0.8	0.3	–	2.2	0.623	0.1	0.0	–	1.1	0.067
	Other^e^	38/94	40.4	2.3	1.3	–	3.8	**0.002**	2.1	1.2	–	3.7	**0.012**
*Drug use*^*f*^	No	217/710	30.6			Ref					Ref		
	Yes	14/85	16.5	0.5	0.2	–	0.8	**0.011**	0.3	0.1	–	0.5	**0.001**
	*CD4 category (cells/mm*^*3*^*)*^*g*^												
	< 200	12/63	19.1			Ref					Ref		
	200–499	136/434	31.3	2.0	1.0	–	4.0	**0.043**	2.3	1.1	–	5.1	**0.032**
	≥ 500	84/299	28.1	1.8	0.9	–	3.6	0.100	2.3	1.0	–	5.1	**0.040**

*aOR* adjusted odds ratio, *OR* crude odds ratio, *CI* confidence interval, *Ref*. reference. Statistically significant* P* values (≤ 0.05) are in bold

^a^Data of 233 participants with a positive antibody test in the first sample (prevalent cases) was analyzed

^b^Row percentages are shown

^c^Includes municipalities in other states

^d^Includes arterial hypertension, diabetes mellitus, asthma, chronic obstructive pulmonary disease, overweight/obesity, cardiovascular diseases, tuberculosis, liver, kidney and autoimmune diseases

^e^Includes bus rapid transit (metrobus), bus, minibus, trolleybus

^f^Drugs other than alcohol, tobacco and intravenous drugs

^g^From the last available blood sample

No effects of consumption of alcohol or tobacco products were observed. Persons vaccinated with CoronaVac vaccine had higher odds of being prevalent cases compared to non-vaccinated persons (*p* = 0.004). In this case it is not possible to differentiate between antibodies produced by vaccination or the infection itself, given that this vaccine is composed of complete inactivated virus. Regarding HIV-associated variables, higher CD4 + T cell counts (200–499 cells/mm^3^: aOR = 2.3, 1.1–5.1, *p* = 0.03; ≥ 500 cells/mm^3^: aOR = 2.3, 1.0–5.1, *p* = 0.04) were associated with higher odds of having a positive serological test at baseline (Table 4). No effect of type of ART regimen or nadir CD4 + T cell count was observed.

### SARS-CoV-2 incidence and associated risks

**Fig. 3:**
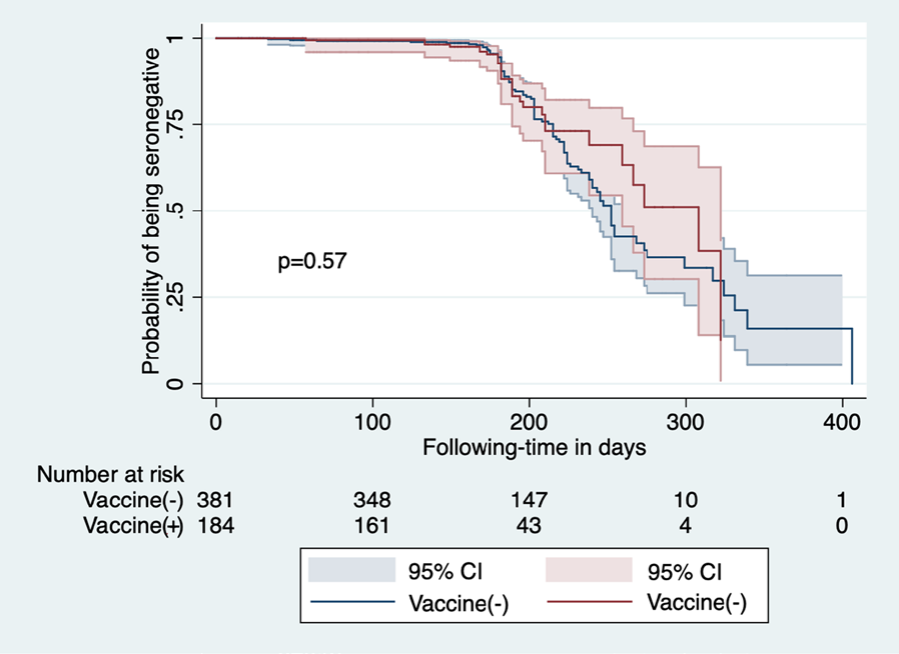
Kaplan–Meier curve assessing the effect of vaccination in incident cases. Only individuals with SARS-CoV-2 negative test in sample 1 were included in the analysis. Follow-up time was measured from donation of sample 1. All individuals included had a previous negative result in sample 1 and reported being vaccinated between sample1 and sample 2. Vaccination was wide available for people living with HIV since march 2021; all samples included in the analysis were obtained after vaccination

The incidence rate in PLWHIV participating in the study was 3.78 cases per 100 person-months. The total person-time follow up was 3493 person-months. We assessed hazards of being an incident case, considering only susceptible individuals (those with a negative test result in sample 1, using a Cox model (Table 5). Persons living in the east zone of Mexico City compared to the south had marginally higher hazard of being incident cases (aHR = 1.9, 95% CI 1.0–3.5; *p* = 0.045). Age, sex, and the presence of comorbidities were not associated with incidence. Regarding community-associated risk, neither attending meetings without social distancing nor the use of public transport were associated with higher risk of being an incident case. Importantly, having been vaccinated with Pfizer/BioNTech(BNT162b2) vaccine was protective compared to not having received any vaccine (aHR = 0.4, 0.2–0.8; *p* = 0.013). No HIV-associated variables (including CD4 + T cell count, CD4 + T cell nadir, CD4:CD8 ratio, time with suppressed viral load, type of ART regimen) showed an effect on being an incident case. Using a Kaplan–Meier curve, no significant overall differences in incidence were observed between vaccinated and non-vaccinated participants (Fig. 3).

**Table 5:** Cox model on risk associations with being a SARS-CoV-2 incident case in study participants.^a^

		HR		95% CI		*p* value	aHR		95% CI		*p* value
Age (years)		0.99	0.97	–	1.00	0.148	1.00	0.98	–	1.03	0.572
Vaccinated against COVID-19	No			Ref					Ref		
	Yes	0.9	0.6	–	1.4	0.657	1.2	0.7	–	1.9	0.553
Municipality	South Mexico City			Ref					Ref		
	North Mexico City	1.3	0.6	–	2.4	0.505	1.3	0.6	–	2.8	0.440
	East Mexico City	1.6	0.9	–	2.9	0.079	1.9	1.0	–	3.5	**0.045**
	West Mexico City	1.4	0.8	–	2.3	0.237	1.6	0.9	–	2.8	0.105
	State of Mexico	1.2	0.7	–	2.0	0.575	1.3	0.7	–	2.2	0.435
	Other^b^	1.0	0.5	–	2.0	0.954	0.8	0.4	–	1.7	0.553
Comorbidities^c^	No			Ref					Ref		
	Yes	1.0	0.6	–	1.5	0.889	0.9	0.6	–	1.4	0.697
Meetings without social distancing	No			Ref					Ref		
	Yes	1.1	0.7	–	1.5	0.756	1.1	0.7	–	1.6	0.728
Use of public transport	No			Ref					Ref		
	Subway	1.0	0.7	–	1.4	0.948	0.8	0.5	-	1.2	0.279
	Taxi/Uber	0.8	0.3	–	2.3	0.702	0.8	0.3	–	2.4	0.702
	Other^d^	1.3	0.8	–	2.3	0.285	1.1	0.6	–	2.1	0.657
Type of COVID-19 vaccine	Non-vaccinated			Ref					Ref		
	BTN162b2 (Pfizer/BioNTech)	0.4	0.2	–	0.8	**0.005**	0.4	0.2	–	0.8	**0.013**
	AZD1222 (AstraZeneca)	1.0	0.6	–	1.4	0.866	1.0	0.6	–	1.6	0.858
	Ad5-nCoV (CanSino)	1.4	0.6	–	3.1	0.380	1.7	0.7	–	4.0	0.239
	Gam-COVID-Vac (Sputnik-V)	0.7	0.4	–	1.6	0.432	0.6	0.3	–	1.5	0.280
	CoronaVac (Sinovac)	1.3	0.5	–	3.6	0.622	1.2	0.4	–	3.7	0.805
CD4 category (cells/mm^3^)^e^	< 200			Ref					Ref		
	200–499	0.9	0.5	–	1.8	0.813	0.9	0.5	–	1.8	0.798
	≥ 500	1.0	0.5	–	2.0	0.968	1.1	0.5	–	2.5	0.727
CD4:CD8 ratio^e^		0.9	0.6	–	1.4	0.653	0.9	0.5	–	1.6	0.732
Time with suppressed VL (years)		1.0	0.9	–	1.0	0.319	1.0	0.9	–	1.0	0.436

*HR* crude hazard ratio, *aHR* adjusted hazard ratio, *CI* confidence interval, *Ref.* reference, *VL* viral load. Statistically significant* P* values (≤ 0.05) are in bold

^a^Prevalent cases (n = 233) were excluded from the model

^b^Includes municipalities in other states; c Includes arterial hypertension, diabetes mellitus, asthma, chronic obstructive pulmonary disease, overweight/obesity, cardiovascular diseases, tuberculosis, liver, kidney and autoimmune diseases^d^Includes bus rapid transit (metrobus), bus, minibus and trolleybus

^e^From the last available blood sample

### SARS-CoV-2 neutralizing antibody titers

**Fig. 4:**
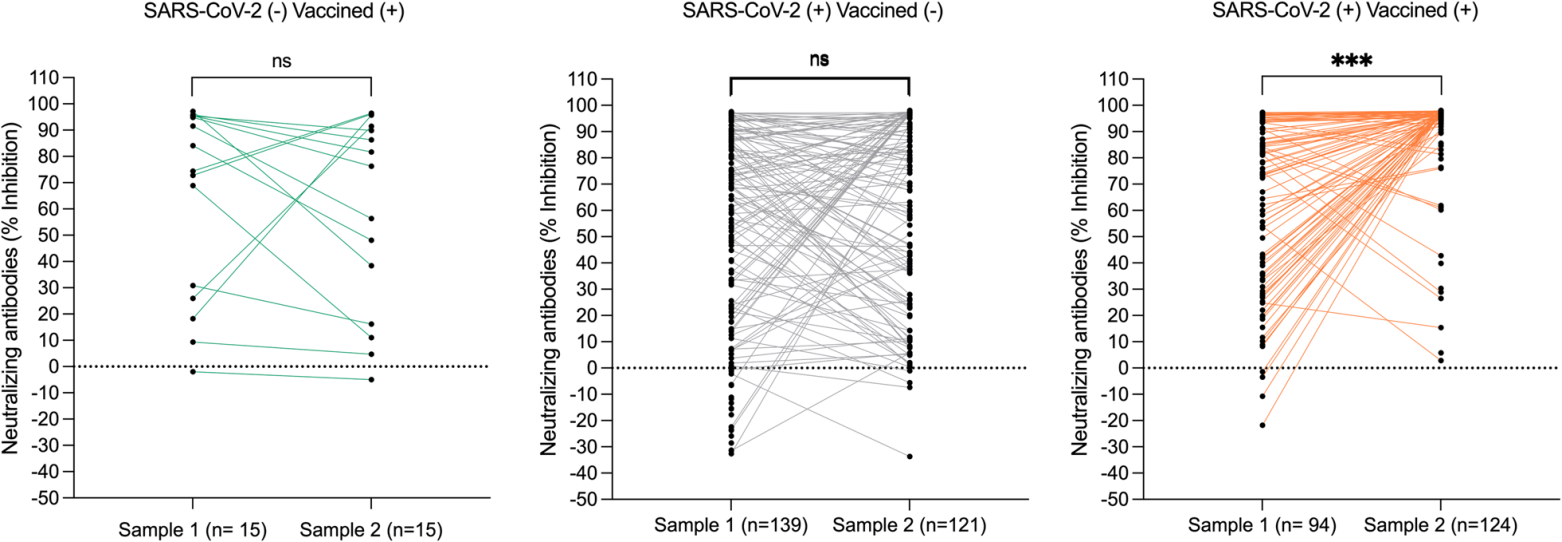
Changes in neutralizing antibody titers with time in PLWHIV on virological suppression. Neutralizing activity was compared between the first and second sample for each participant and expressed as % inhibition using a surrogate virus neutralization test. Participants split into three groups: unvaccinated persons with previous SARS-CoV-2 infection (assessed by a positive total anti-N antibody test), displayed in green; vaccinated persons without previous SARS-CoV-2 infection, displayed in gray; and previously SARS-CoV-2 infected and vaccinated person, displayed in orange; ***p < 0.0001; ns, non-statistically significant

**Fig. 5:**
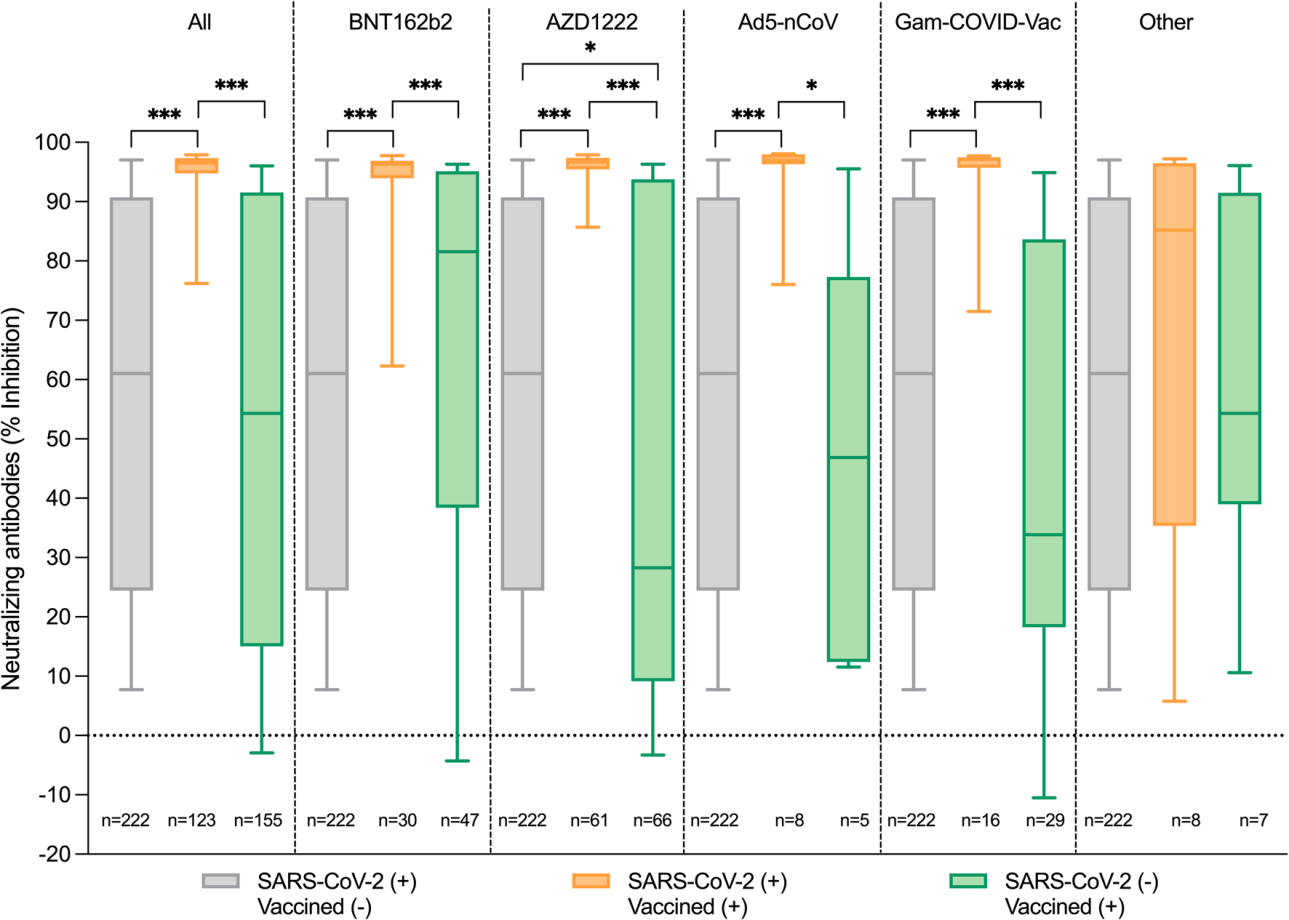
Effect of SARS-CoV-2 vaccine type in PLWHIV on virological suppression. Neutralizing antibody activity measured as % inhibition in a surrogate virus neutralization test was compared for four of the locally approved and available vaccines in Mexico in unvaccinated persons with previous SARS-CoV-2 infection, vaccinated persons without previous SARS-CoV-2 infection, and previously SARS-CoV-2 infected and vaccinated persons. Other vaccines include: Moderna (Spikevax), Janssen (Ad26.COV2-5), and SinoVac (CoronaVac). Significant differences between groups are indicated. *p < 0.01, **p < 0.001, ***p < 0.0001

We studied SARS-CoV-2 neutralizing antibody titers in PLWHIV in three scenarios: Persons who were vaccinated and had no evidence of previous SARS-CoV-2 infection [SARS-CoV-2(−)/Vaccine(+)], Non-vaccinated persons with previous SARS-CoV-2 infection [SARS-CoV-2(+)/Vaccine(−)], and vaccinated persons with previous SARS-Cov-2 infection [SARS-CoV-2(+)/Vaccine(+)]. Comparing the first and second sample, with a median time between samples of 187 days (IQR 172–208), neutralizing antibody titers remained overall similar in the SARS-CoV-2(−) Vaccine(+) and the SARS-CoV-2(+)/Vaccine(−) groups. However, a marked increase in neutralizing titers was observed in SARS-CoV-2(+)/Vaccine(+) persons (*p* < 0.001) (Fig. 4) with maximization of neutralizing activity in most participants of this group (Additional file 3). This increase was significant for the Pfizer/BioNTech(BNT162b2), AstraZeneca(AZD1222), CanSino(Ad5-nCoV), and Sputnik-V(Gam-CoVID-Vac) vaccines (Fig. 5). Comparing the SARS-Cov-2(+)Vaccine(−) and the SARS-CoV-2(−)Vaccine(+) groups, no significant differences in neutralizing antibody titers were observed except for the AstraZeneca AZD1222 group, which showed lower titers in vaccinated only vs. infected only individuals (Fig. 5). The number of participants with other types of vaccines, including Moderna(Spikevax), Janssen(Ad26.COV2-5), and SinoVac(CoronaVac) was too small to obtain reliable data.

In order to further evaluate the role of vaccination in prevalent cases, we assessed the effect of time after vaccination on neutralizing activity (Additional file 4). The median time between the positive antibodies test result and the reported date of vaccination was 114 (IQR: 77–149) days. To assess it, we subtracted the date of vaccination to the date of the first sample donation, thus, samples with a negative value correspond to individuals whose sample was donated before vaccination. Based on their serostatus, samples split in two groups: positive anti-N antibodies [SARS-CoV-2(+)] and negative anti-N antibodies [SARS-CoV-2(−)], demonstrating that persons that received the vaccine and had previous SARS-CoV-2 infection had higher inhibition percentage than those who received the vaccine and had not been infected.

We next explored possible associations between SARS-CoV-2 neutralizing antibody titers in previously COVID-19-vaccinated participants without evidence of previous SARS-CoV-2 infection, using a linear regression model with neutralization percentage as the dependent variable (Additional file 5). Higher neutralization was associated with higher age (adjusted coefficient: 0.7; *p* = 0.045), and lower time between vaccination and the last sample included in the study (adjusted coefficient: − 0.2; *p* = 0.017). Associations with different types of COVID-19 vaccines were lost after multivariable adjustment. No associations with CD4 + T cell count, CD4 + T cell nadir, CD4:CD8 ratio or time with suppressed VL were observed.

## Discussion

In this work, we assessed SARS-CoV-2 antibody prevalence and titers, as well as individual and community risks associated with SARS-CoV-2 infection in a group of PLWHIV on ART, cared for at a large tertiary referral center in Mexico City. We observed that SARS-CoV-2 infection in the study group was associated with community risk, rather than HIV-associated clinical variables. Indeed, younger age, place of residence out of Mexico City, use of public transport, and attending meetings without social distancing were associated with higher odds of being a prevalent case. Higher CD4 + T cell counts (> 200 cells/mm^3^) were also associated with higher odds of a positive baseline serological test. Although this observation may seem counterintuitive, as persons with ≤ 200 cells/mm^3^ have been considered a higher risk population [10], it might be explained by greater caution in the more immunosuppressed participants to avoid SARS-CoV-2 exposure, or bias due to underrepresentation in the study. Thus, our analyses suggest a similar risk of SARS-CoV-2 infection in PLWHIV under viral suppression, independently of their current CD4 + T cell count, time on ART or nadir CD4 + T cell count.

We observed an overall adjusted prevalence of 45.57% that suggests a high level of community-associated exposure in Mexico City. This study was conducted mostly during the third wave of the COVID-19 epidemic in Mexico, dominated by the Delta variant [16]. The estimated prevalence in Mexico after the second wave, in late 2020, was reported to be 35.9% [17] and 33.5% in healthcare personnel in Mexico City by mid-2021 [18]. This is consistent with the higher prevalence observed in this study, given the expected increase in exposure associated with the Delta wave in 2021, as well as the gradual return to normal economic and social activities (including opening of schools), with a significant increase in mobility [5, 19].

SARS-CoV-2 case-distribution has been uneven within Mexico City [20]. We observed higher odds of being a prevalent case in the Western region, as well as an increased hazard of being an incident case in the Eastern region. During the study period, the Western region, had the highest cumulative case rate in Mexico City, while the cumulative case rate increased more than 40% between June and September 2021 in the Eastern region [20]. These results correspond to the most densely populated geographic areas [21], but not necessarily to the areas with the highest HIV prevalence in Mexico City [22]; SARS-CoV-2 prevalence and incidence were modified during the course of the pandemic with the implementation of preventive measures, and the progression in the vaccination strategy, highlighting the major role of community exposure to SARS-CoV-2, which does not seem to be related to HIV prevalence.

We observed higher odds of being a prevalent case in users of public transportation other than the subway (Table 4). This observation coincides with a larger increase in mobility by trolleybus (compared to the subway) during the study period [23]. Mobility becomes especially relevant in the epidemiological context of Mexico City’s metropolitan area, with characteristically long and crowded commutes [19].

In our study, older participants had lower odds of being seropositive, which is in agreement with the reported higher incidence in people younger than 50 years in the same period [20]. The protective effect of higher age observed in our study could be related to the vaccination strategy in Mexico, as older persons were prioritized to receive the COVID-19 vaccine first [24]. Also, the higher proportion of cases in younger people (Table 1) may be related to the age structure of the Mexican population (with predominance of people in productive age), the confinement of high-risk groups (including the elderly), the return to occupational and recreational activities, and lower uptake of lockdown and prevention measures in the younger population.

Despite the high vaccination coverage in Mexico City [6], a correlation between vaccination and lower risk of SARS-CoV-2 infection was only evident for the BTN162b2(Pfizer/BioNTech) vaccine in adjusted models. This suggests that this vaccine could perhaps confer better protection against infection. Nevertheless, even when no differences were observed in the completeness of vaccination regimens for the different vaccines used in the study population, uneven exposure to SARS-CoV-2 could have occurred between vaccine groups, given the differences found in age and geographic location (Additional file 6). Moreover, the lack of protection against infection is not surprising given that the main effect of COVID-19 vaccines is prevention of severe disease and death [25, 26]. Moreover, effects of other vaccines could have been missed because of statistical power issues. Regardless, improvement in vaccination coverage as prevalence of natural SARS-CoV-2 infection increases, may lead to beneficial changes in the pandemic dynamics, with less severe outcomes [27], and through a likely synergistic immune response in people that have been vaccinated and infected [26], as also seen in the present study.

Our data contrast with the prevalence of obesity (31.5%) reported for Mexico City in 2020, as well as with the previously reported association of higher SARS-CoV-2 prevalence in people with obesity in the Mexican population [28], which may suggest enrolment bias due to more strict confinement of persons with higher risk of severe disease. Additionally, information bias due to underestimation of chronic diseases in our study may exist, as this information was self-reported and the screening of chronic diseases in PLWHIV was remarkably reduced during the pandemic [7].

Surprisingly, drugs different from alcohol, tobacco or intravenous drugs were associated with lower odds of being a prevalent case. As most participants did not specify the drugs consumed, we cannot make solid inferences about this protective effect. Nonetheless, cannabis is the most frequently consumed drug after alcohol and tobacco in Mexico [29]. Overall, substance abuse has been associated with higher prevalence or severity of SARS-CoV-2 for most drugs, including cocaine, opioids, stimulants, and sedatives. In contrast, cannabinoids have been hypothesized to have positive effects due to their immunomodulatory and anti-inflammatory actions, as well as the possible blocking of viral entry to the host cell [30], while the effect of inhaled cannabis remains controversial [31, 32].

Our observations are limited to a sample of PLWHIV on virological control and good clinical follow-up in a reference institution that was able to continue access to HIV clinical services (although with restrictions) and guarantee continuity of ART, using strategies such as telemedicine, multi-month dispensing of antiretrovirals, and mental health follow-up teleconsultations. These results do not necessarily reflect risks in other scenarios occurring in the country, with possible HIV service disruptions, and treatment interruptions. Moreover, the design of our study did not allow us to assess risks of mortality or more severe COVID-19 in PLWHIV on virological control. Overall, our observations suggest that risk of SARS-CoV-2 infection in PLWHIV engaged to care and on virological suppression are community-related and do not reflect immune status. Similarly, antibody response toward SARS-CoV-2 infection and vaccination was comparable to that of the general population and was not associated with immune status.

## Conclusions

SARS-CoV-2 infection prevalence in the studied population of PLWHIV on ART with good clinical follow-up was high and similar to that expected in the general population after the third wave of the COVID-19 epidemic in Mexico. SARS-CoV-2 infection in this group was associated with community risk, rather than HIV-associated clinical variables. SARS-CoV-2 antibody neutralization activity in vaccinated persons was maximized with previous SARS-CoV-2 infection and was not associated with CD4 + T cell counts or time with suppressed viral load. Our study suggests, that PLWHIV under ART and viral control have similar risk of exposure to the general population and that antibody titers are not associated with clinical status. Further studies are needed to assess the quality of immune responses in this population.

### Supplementary Information

**Additional file 1:** Clinical and behavioral characteristics of PLWHIV participating in the study.

**Additional file 2:** COVID-19 associated symptoms report in PLWHIV participating in the study.

**Additional file 3:** Correlation between total anti-N and neutralizing SARS-CoV-2 antibodies in PLWHIV on virologic suppression.

**Additional file 4:** Correlations between SARS-CoV-2 neutralization activity and time from vaccination.

**Additional file 5:** Associations with SARS-CoV-2 neutralizing antibody titters in vaccinated PLWHIV participating in the study.

**Additional file 6:** Characteristics of participants by type of SARS-CoV-2 vaccine.

## Data Availability

The datasets used and/or analysed during the current study are available from the corresponding author on reasonable request.
