# Supplementary material for: SARSCoV-2 antibody prevalence and titers in persons living with HIV cared for at a large tertiary reference center in Mexico City
Source: Virol J. 2023 Dec 15;20:300. doi: 10.1186/s12985-023-02261-2 (PMC10724955; doi:10.1186/s12985-023-02261-2)
Supplement: Supplementary file 1 — Additional file 1: Clinical and behavioral characteristics of PLWHIV participating in the study. [file 12985_2023_2261_MOESM1_ESM.docx]

| Additional File 1. Clinical and behavioral characteristics of PLWHIV participating in the study. | | | | | | | |  |  |  |
| --- | --- | --- | --- | --- | --- | --- | --- | --- | --- | --- |
|  |  |  | **Prevalent cases (n=233)** |  |  | **Incident cases (n=132)** |  |  | **Non-cases (n=437)** |  |
|  |  | **n** | **%^a^** | ***p* value^b^** | **n** | **%^a^** | ***p* value^b^** | **n** | **%^a^** | ***p* value^b^** |
| Current ART regimen | BIC/TAF/FTC | 176 | 75.5 | 0.991 | 103 | 79.8 | 0.122 | 323 | 73.9 | 0.300 |
|  | EFV/TDF/FTC | 29 | 12.5 |  | 17 | 13.2 |  | 53 | 12.1 |  |
|  | Other^c^ | 28 | 12.0 |  | 9 | 7.0 |  | 61 | 14.0 |  |
| Continuted ART during the COVID-19 pandemic | Yes | 228 | 97.9 | 0.776 | 128 | 97.7 | 0.735 | 429 | 98.4 | 0.605 |
|  | No | 5 | 2.2 |  | 3 | 2.3 |  | 7 | 1.6 |  |
| Medications other than antiretrovirals^d^ | Yes | 56 | 24.0 | 0.478 | 33 | 25.0 | 0.913 | 118 | 27.0 | 0.466 |
|  | No | 177 | 76.0 |  | 99 | 75.0 |  | 319 | 73.0 |  |
| Comorbidities^e^ | Yes | 66 | 28.3 | 0.662 | 31 | 23.5 | 0.337 | 120 | 27.5 | 0.873 |
|  | No | 167 | 71.7 |  | 101 | 76.5 |  | 317 | 72.5 |  |
| Alcohol consumption | Daily | 2 | 0.9 | 0.582 | 1 | 0.8 | 0.602 | 5 | 1.1 | 0.298 |
|  | Weekly | 17 | 7.3 |  | 10 | 7.6 |  | 35 | 8.0 |  |
|  | Monthly | 21 | 9.0 |  | 8 | 6.1 |  | 29 | 6.6 |  |
|  | Occasionally | 106 | 45.5 |  | 66 | 50.0 |  | 177 | 40.5 |  |
|  | No | 87 | 37.3 |  | 47 | 35.6 |  | 191 | 43.7 |  |
| Consumption of tobacco-derived products | Heated tobacco products^f^ | 1 | 0.4 | 0.170 | 0 | 0 | 0.853 | 2 | 0.5 | 0.209 |
|  | Electronic cigarette | 2 | 0.9 |  | 2 | 1.5 |  | 4 | 0.9 |  |
|  | Cigarette | 50 | 21.5 |  | 34 | 25.8 |  | 127 | 29.1 |  |
|  | No | 180 | 77.3 |  | 96 | 72.7 |  | 304 | 69.6 |  |
| Consumption frequency of tobacco products | Daily | 25 | 10.7 | 0.195 | 16 | 12.1 | 0.783 | 71 | 16.3 | 0.222 |
|  | Weekly | 6 | 2.6 |  | 7 | 5.3 |  | 17 | 3.9 |  |
|  | Monthly | 2 | 0.9 |  | 2 | 1.5 |  | 7 | 1.6 |  |
|  | Occasionally | 36 | 15.5 |  | 21 | 15.9 |  | 71 | 16.3 |  |
|  | Never | 164 | 70.4 |  | 86 | 65.2 |  | 271 | 62.0 |  |
| Intravenous drug use | Yes | 1 | 0.4 | 0.405 | 2 | 1.5 | 0.432 | 5 | 1.1 | 0.478 |
|  | No | 230 | 98.7 |  | 129 | 97.7 |  | 431 | 98.6 |  |
|  | Prefer not to answer | 2 | 0.9 |  | 1 | 0.8 |  | 1 | 0.2 |  |
| Use of other drugs^g^ | Yes | 14 | 6.0 | **0.004** | 17 | 12.9 | 0.370 | 54 | 12.4 | 0.119 |
|  | No | 217 | 93.1 |  | 114 | 86.4 |  | 382 | 87.4 |  |
|  | Unknown/Prefer not to answer | 2 | 0.9 |  | 1 | 0.8 |  | 1 | 0.2 |  |
| Meetings without social distancing | Yes | 101 | 43.4 | 0.342 | 55 | 41.7 | 0.846 | 170 | 38.9 | 0.248 |
|  | No | 132 | 56.7 |  | 77 | 58.3 |  | 267 | 61.1 |  |
| Use of face mask | Always | 195 | 83.7 | 0.127 | 101 | 76.5 | 0.234 | 353 | 80.8 | 0.165 |
|  | Generally | 33 | 14.2 |  | 28 | 21.2 |  | 81 | 18.5 |  |
|  | Sometimes | 5 | 2.2 |  | 3 | 2.3 |  | 3 | 0.7 |  |
| Hand washing | Always | 187 | 80.3 | 0.314 | 106 | 80.3 | 0.868 | 357 | 81.7 | 0.558 |
|  | Generally | 36 | 15.5 |  | 23 | 17.4 |  | 70 | 16.0 |  |
|  | Sometimes | 10 | 4.3 |  | 3 | 2.3 |  | 10 | 2.3 |  |
| Social distancing | Always | 156 | 67.0 | 0.177 | 89 | 67.4 | 0.900 | 275 | 62.9 | 0.351 |
|  | Generally | 57 | 24.5 |  | 32 | 24.2 |  | 124 | 28.4 |  |
|  | Sometimes | 17 | 7.3 |  | 11 | 8.3 |  | 37 | 8.5 |  |
|  | Never | 3 | 1.3 |  | 0 | 0 |  | 1 | 0.2 |  |
| Contact with any person with COVID-19 | Yes | 106 | 45.5 | **0.001** | 53 | 40.2 | **0.008** | 147 | 33.6 | **0.016** |
|  | No | 113 | 48.5 |  | 55 | 41.7 |  | 240 | 54.9 |  |
|  | Unknown | 14 | 6.0 |  | 24 | 18.2 |  | 50 | 11.4 |  |
| COVID-19 symptoms requiring medical care | Yes | 84 | 36.1 | **0.003** | 55 | 41.7 | **0.001** | 90 | 20.6 | **0.001** |
|  | No | 149 | 64.0 |  | 77 | 58.3 |  | 347 | 79.4 |  |
| Supplemental oxygen requirement | Yes | 8 | 3.4 | 0.920 | 7 | 5.3 | **0.009** | 1 | 0.2 | **0.001** |
|  | No | 225 | 96.6 |  | 125 | 94.7 |  | 436 | 99.8 |  |
| Hospitalization requirement | Yes | 7 | 3.0 | 0.048 | 4 | 3.0 | 0.118 | 1 | 0.2 | **0.002** |
|  | No | 226 | 97.0 |  | 128 | 97.0 |  | 436 | 99.8 |  |
| ART, antiretroviral treatment; BIC, bictegravir; TAF, tenofovir alafenamide; TDF, tenofovir disoproxyl fumarate; FTC, emtricitabine; EFV, efavirenz; IV, intravenous; ^a^ Column percentages are shown; ^b^ Fisher exact test, two-sided p values are shown comparing each group against the rest of the participants; ^c^ Includes ART regimens containing etravirine, bictegravir, darunavir, dolutegravir and efavirenz; ^d^ Includes steroids (oral or inhaled), blood pressure medications, diabetes medications, pain medications, cancer medications and antidepressants; ^e^ Includes arterial hypertension, diabetes mellitus, asthma, chronic obstructive pulmonary disease, overweight/obesity, cardiovascular diseases, tuberculosis, liver, kidney and autoimmune diseases; ^f^ Includes iQOS/Ploom, Glo and PAX; ^g^ Drugs other than alcohol, tobacco and intravenous drugs. | | | | | | | | | | |
