# Supplementary material for: SARSCoV-2 antibody prevalence and titers in persons living with HIV cared for at a large tertiary reference center in Mexico City
Source: Virol J. 2023 Dec 15;20:300. doi: 10.1186/s12985-023-02261-2 (PMC10724955; doi:10.1186/s12985-023-02261-2)
Supplement: Supplementary file 2 — Additional file 2: COVID-19 associated symptoms report in PLWHIV participating in the study. [file 12985_2023_2261_MOESM2_ESM.docx]

| Additional file 2. COVID-19 associated symptoms report in PLWHIV participating in the study.^a^ | | | | | | | |
| --- | --- | --- | --- | --- | --- | --- | --- |
| Symptom | **Prevalent cases (n=233)** | | **Incident cases (n=132)** | | **Non-cases (n=437)** | |  |
|  | **n** | **%^b^** | **n** | **%^b^** | **n** | **%^b^** | ***p* value^c^** |
| Fever | 65 | 27.9 | 51 | 38.6 | 59 | 13.5 | **< 0.001** |
| Chills | 72 | 30.9 | 53 | 40.2 | 70 | 16 | **< 0.001** |
| Fatigue | 109 | 46.8 | 72 | 54.6 | 144 | 33 | **< 0.001** |
| Myalgia | 89 | 38.2 | 67 | 50.8 | 111 | 25.4 | **< 0.001** |
| Sore throat | 79 | 33.9 | 51 | 38.6 | 96 | 22 | **< 0.001** |
| Cough | 76 | 32.6 | 53 | 40.2 | 91 | 20.8 | **< 0.001** |
| Rhinorrhea | 70 | 30 | 53 | 40.2 | 98 | 22.4 | **< 0.001** |
| Dyspnea | 41 | 17.6 | 28 | 21.2 | 34 | 7.8 | **< 0.001** |
| Thoracic pain | 42 | 18 | 28 | 21.2 | 33 | 7.6 | **< 0.001** |
| Other respiratory | 25 | 10.7 | 21 | 15.9 | 24 | 5.5 | **< 0.001** |
| Headache | 86 | 36.9 | 59 | 44.7 | 135 | 30.9 | **0.011** |
| Nausea | 29 | 12.5 | 32 | 24.2 | 38 | 8.7 | **0.001** |
| Abdominal pain | 30 | 12.9 | 31 | 23.5 | 43 | 9.8 | **0.004** |
| Diarrhea | 44 | 18.9 | 38 | 28.8 | 86 | 19.7 | 0.338 |
| Anosmia | 75 | 32.2 | 46 | 34.9 | 20 | 4.6 | **< 0.001** |
| Dysgeusia | 69 | 29.6 | 42 | 31.8 | 19 | 4.4 | **< 0.001** |
| ^a^ Self-reported symptoms since the beginning of the pandemic (March 2020); ^b^ Column percentages are shown; ^c^Fisher’s exact test, two-sided p values are shown comparing all positive cases (prevalent cases + incident cases) vs non-cases. | | | | | | | |
