# Supplementary material for: SARSCoV-2 antibody prevalence and titers in persons living with HIV cared for at a large tertiary reference center in Mexico City
Source: Virol J. 2023 Dec 15;20:300. doi: 10.1186/s12985-023-02261-2 (PMC10724955; doi:10.1186/s12985-023-02261-2)
Supplement: Supplementary file 3 — Additional file 3: Correlation between total anti-N and neutralizing SARS-CoV-2 antibodies in PLWHIV on virologic suppression. [file 12985_2023_2261_MOESM3_ESM.docx]

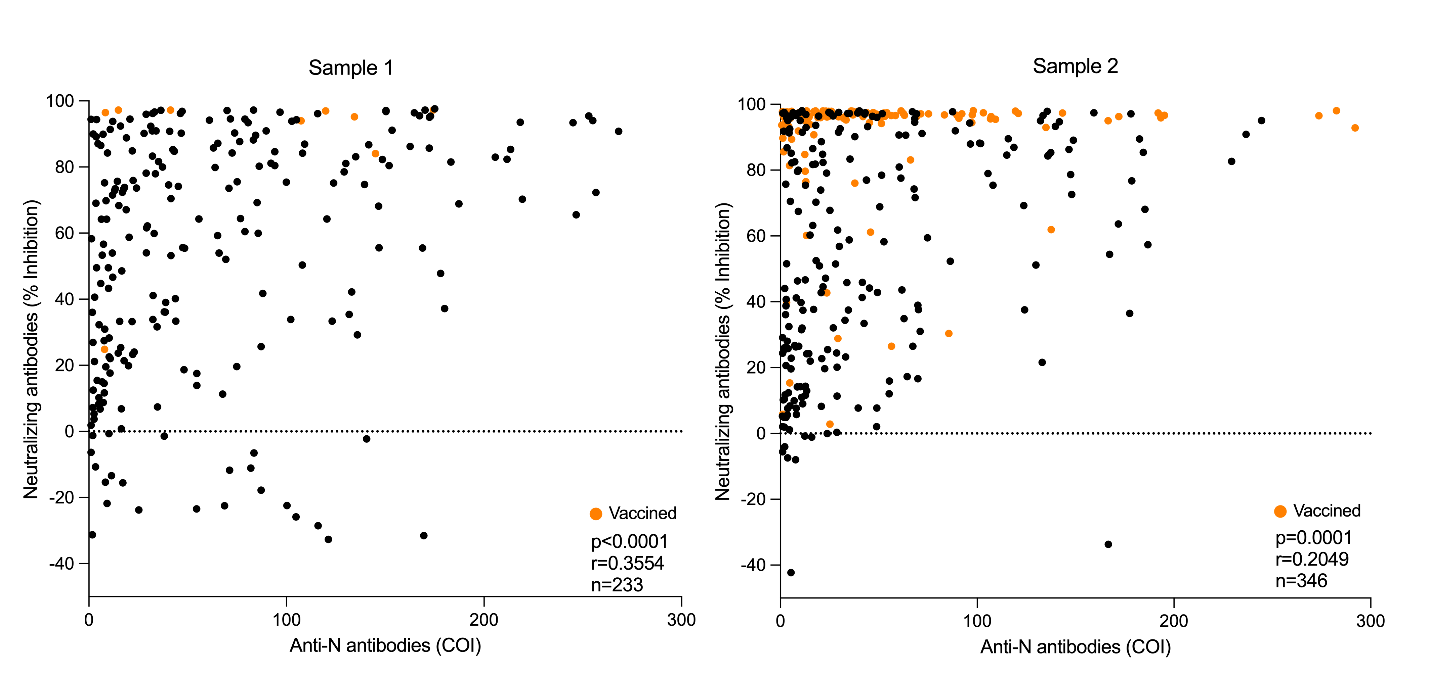


**Additional file 3. Correlation between total anti-N and neutralizing SARS-CoV-2 antibodies in PLWHIV on virologic suppression.** Spearman correlations were performed for sample 1 and sample 2, for all participants with evidence of SARS-CoV-2 infection. Total anti-N antibodies are expressed as cut off index (COI) of an electrochemiluminescence-based commercial assay. Neutralization activity is expressed as % inhibition of a surrogate virus neutralization test. Vaccinated persons (at least one dose of any of the available vaccines) at the time of sample 1 or sample 2 donation are highlighted.
