# Supplementary material for: SARSCoV-2 antibody prevalence and titers in persons living with HIV cared for at a large tertiary reference center in Mexico City
Source: Virol J. 2023 Dec 15;20:300. doi: 10.1186/s12985-023-02261-2 (PMC10724955; doi:10.1186/s12985-023-02261-2)
Supplement: Supplementary file 4 — Additional file 4: Correlations between SARS-CoV-2 neutralization activity and time from vaccination. [file 12985_2023_2261_MOESM4_ESM.docx]

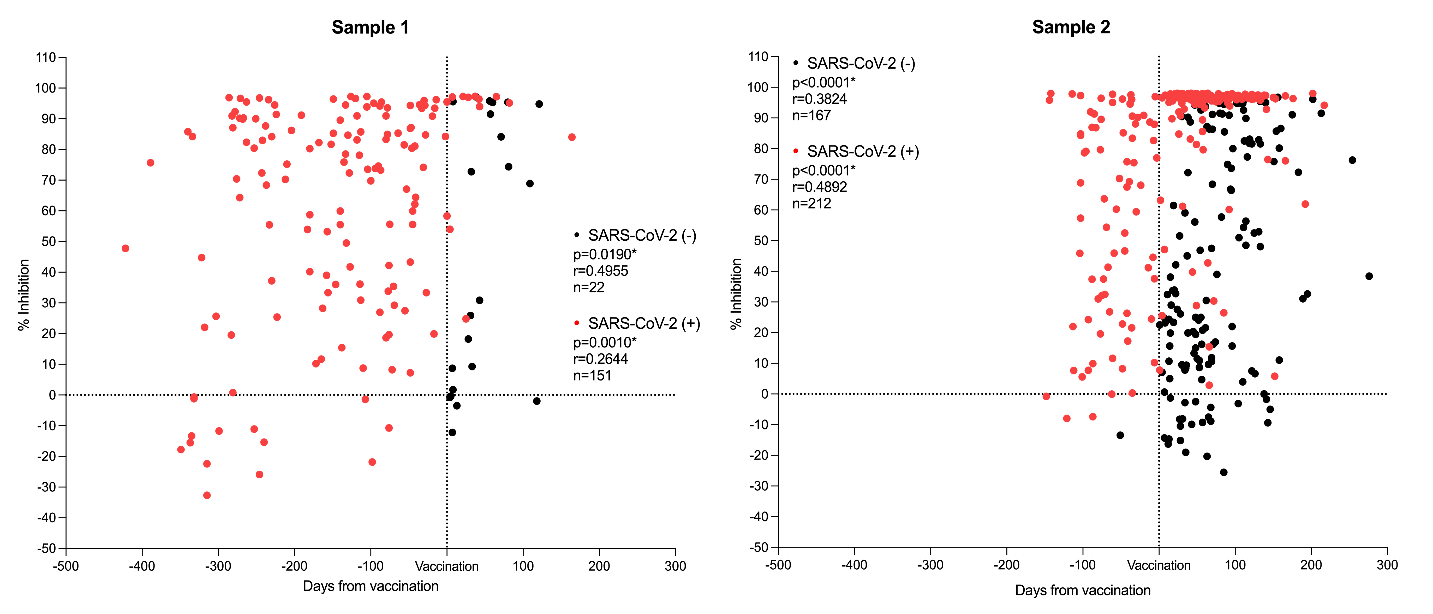


**Additional file 4. Correlations between SARS-CoV-2 neutralization activity and time from vaccination.** Spearman correlations were performed for sample 1 and sample 2, for SARS-CoV-2 (+) samples (displayed in red) and SARS-CoV-2 (-) samples (displayed in black), according to the antibody tests result. Each dot represents a person. Neutralization activity is expressed as % inhibition of a surrogate virus neutralization test. The median times from vaccination to sample 1 and sample 2 donation are shown by vertical lines (negative numbers represent days before vaccination).
