# Supplementary material for: SARSCoV-2 antibody prevalence and titers in persons living with HIV cared for at a large tertiary reference center in Mexico City
Source: Virol J. 2023 Dec 15;20:300. doi: 10.1186/s12985-023-02261-2 (PMC10724955; doi:10.1186/s12985-023-02261-2)
Supplement: Supplementary file 5 — Additional file 5: Associations with SARS-CoV-2 neutralizing antibody titters in vaccinated PLWHIV participating in the study. [file 12985_2023_2261_MOESM5_ESM.docx]

| Additional file 5. Associations with SARS-CoV-2 neutralizing antibody titters in vaccinated PLWHIV participating in the study.^a^ | | | | | | | | | | | | | | | | | | | |
| --- | --- | --- | --- | --- | --- | --- | --- | --- | --- | --- | --- | --- | --- | --- | --- | --- | --- | --- | --- |
|  |  | **Coef.** | | **95% CI** | | | | ***p* value** | | **aCoef.** | | **95% CI** | | | | | ***P***  **value** | |  |
| Age (years) |  | 1.1 | | 0.5 | - | | 1.7 | | **0.001** | | 0.7 | | 0.0 | - | 1.5 | **0.045** | |  |  |
| State of residency | Mexico City | | |  | Ref. | |  | |  | |  | |  | Ref. |  |  | |  |  |
|  | State of Mexico | | -16.6 | -33 | - | | -0.1 | | **0.048** | | -12.1 | | -28.5 | - | 4.3 | 0.146 | |  |  |
|  | Other^b^ | | -27.1 | -53.4 | - | | -0.9 | | **0.043** | | -32 | | -58.0 | - | -6 | **0.016** | |  |  |
| Comorbidities^d^ | No | |  |  | Ref. | |  | |  | |  | |  | Ref. |  |  | |  |  |
|  | Yes | | 7.7 | -6 | - | | 21.4 | | 0.267 | | 3 | | -10.4 | - | 16.5 | 0.654 | |  |  |
| Type of COVID-19 vaccine | BNT162b2 (Pfizer/BioNTech) | | | | | Ref. |  | |  | |  | |  | Ref. |  |  | |  |  |
|  | AZD1222 (AstraZeneca) | | -20.7 | -35.2 | - | | -6.1 | | **0.006** | | -6.5 | | -22.6 | - | 9.5 | 0.423 | |  |  |
|  | Ad5-nCoV (Cansino) | | -19.8 | -55.2 | - | | 15.7 | | 0.273 | | -4.5 | | -40.7 | - | 31.7 | 0.807 | |  |  |
|  | Gam-COVID-Vac (Sputnik V) | | -20.2 | -38 | - | | -2.4 | | **0.026** | | -8.8 | | -27.5 | - | 9.8 | 0.352 | |  |  |
|  | Ad26.COV2-S (Janssen) | | -18.0 | -53.4 | - | | 17.5 | | 0.318 | | -5.4 | | -42.4 | - | 31.7 | 0.775 | |  |  |
|  | Spikevax (Moderna) | | 28.8 | -25.6 | - | | 83.2 | | 0.298 | | 15.8 | | -36.9 | - | 68.5 | 0.555 | |  |  |
| Alcohol consumption | No | |  |  | Ref. | |  | |  | |  | |  | Ref. |  |  | |  |  |
|  | Yes | | 12.0 | -0.4 | - | | 24.5 | | 0.058 | | 6.9 | | -6.1 | - | 19.9 | 0.298 | |  |  |
| Consumption of tobacco-derived products | Non-smoker | | |  | Ref. | |  | |  | |  | |  | Ref. |  |  | |  |  |
|  | Cigarette | | 0.0 | -14.4 | - | | 14.3 | | 0.996 | | -2.3 | | -16.4 | - | 11.7 | 0.743 | |  |  |
|  | Heated tobacco products^d^ | | -9.2 | -87.3 | - | | 68.8 | | 0.815 | | -12.3 | | -88.4 | - | 63.7 | 0.749 | |  |  |
| Other drugs^e^ | No | |  |  | Ref. | |  | |  | |  | |  | Ref. |  |  | |  |  |
|  | Yes | | 11.3 | -9.1 | - | | 31.7 | | 0.274 | | 9.2 | | -11.7 | - | 30.1 | 0.388 | |  |  |
| Time between vaccination and last available sample | N/A | | -0.2 | -0.4 | - | | -0.1 | | **0.001** | | -0.2 | | -0.3 | - | 0.0 | **0.017** | |  |  |
| CD4 category (cells/mm^3^)^f^ | < 200 | |  |  | Ref. | |  | |  | |  | |  | Ref. |  |  | |  |  |
|  | 200-499 | | 9.0 | -11.3 | - | | 29.4 | | 0.381 | | 7 | | -13.1 | - | 27.1 | 0.493 | |  |  |
|  | ≥ 500 | | 23.1 | 1.9 | - | | 44.2 | | **0.033** | | 20.9 | | -1.3 | - | 43.2 | 0.065 | |  |  |
| CD4:CD8 ratio^f^ |  | | 8.7 | -4.9 | - | | 22.3 | | 0.210 | | -6.4 | | -21.3 | - | 8.6 | 0.402 | |  |  |
| Time with suppressed  VL (years) | | | 1.1 | -0.5 | - | | 2.7 | | 0.161 | | -0.3 | | -2.0 | - | 1.5 | 0.772 | |  |  |
| Coef., crude linear regression coefficient; aCoef., adjusted coefficient; CI, confidence interval; Ref., reference; VL, viral load; ^a^ Only vaccinated participants without evidence of prior exposure to SARS-CoV-2 were included; ^b^ Includes other states of Mexico; ^c^ Includes arterial hypertension, diabetes mellitus, asthma, chronic obstructive pulmonary disease, overweight/obesity, cardiovascular diseases, tuberculosis, liver, kidney and autoimmune diseases; ^d^ Includes iQOS/Ploom, Glo and PAX; ^e^ Drugs other than alcohol, tobacco and IV drugs; ^f^ From the last available sample. | | | | | | | | | | | | | | | | | | | |
