# Supplementary material for: SARSCoV-2 antibody prevalence and titers in persons living with HIV cared for at a large tertiary reference center in Mexico City
Source: Virol J. 2023 Dec 15;20:300. doi: 10.1186/s12985-023-02261-2 (PMC10724955; doi:10.1186/s12985-023-02261-2)
Supplement: Supplementary file 6 — Additional file 6: Characteristics of participants by type of SARS-CoV-2 vaccine. [file 12985_2023_2261_MOESM6_ESM.docx]

| Additional file 6. Characteristics of participants by type of SARS-CoV-2 vaccine. | | | | | | | | | | | | | | | | | | | |
| --- | --- | --- | --- | --- | --- | --- | --- | --- | --- | --- | --- | --- | --- | --- | --- | --- | --- | --- | --- |
|  |  | BNT162b2  (Pfizer/BioNTech) | | AZD1222  (AstraZeneca) | | Ad5-nCoV  (Cansino) | | Gam-COVID-Vac  (Sputnik V) | | CoronaVac  (Sinovac) | | Ad26.COV2-S  (Janssen) | | Spikevax  (Moderna) | |  |  |  |  |
|  |  | **n** | **%^a^** | **n** | **%^a^** | **n** | **%^a^** | **n** | **%^a^** | **n** | **%^a^** | **n** | **%^a^** | **n** | **%^a^** | **Total** | **p-value^g^** |  |  |
| Age (years) | < 50 | 26 | 16.4 | 95 | 59.8 | 13 | 8.2 | 17 | 10.7 | 3 | 1.9 | 4 | 2.5 | 1 | 0.6 | 159 | **0.001** |  |  |
|  | ≥ 50 | 51 | 42.9 | 33 | 27.7 | 0 | 0 | 28 | 23.5 | 3 | 2.5 | 1 | 0.8 | 3 | 2.5 | 119 |  |  |  |
| Gender | Cisgender Men | 65 | 27.9 | 107 | 45.9 | 11 | 4.7 | 37 | 15.9 | 5 | 2.2 | 5 | 2.2 | 3 | 1.3 | 233 | 0.992 |  |  |
|  | Cisgender Women | 12 | 27.9 | 19 | 44.2 | 2 | 4.7 | 8 | 18.6 | 1 | 2.3 | 0 | 0 | 1 | 2.3 | 43 |  |  |  |
|  | Transgender Women | 0 | 0 | 1 | 100 | 0 | 0 | 0 | 0 | 0 | 0 | 0 | 0 | 0 | 0 | 1 |  |  |  |
|  | Non-binary | 0 | 0 | 1 | 100 | 0 | 0 | 0 | 0 | 0 | 0 | 0 | 0 | 0 | 0 | 1 |  |  |  |
| Municipality | North Mexico City | 1 | 4.0 | 8 | 32.0 | 1 | 4.0 | 14 | 56.0 | 1 | 4.0 | 0 | 0 | 0 | 0 | 25 | **0.001** |  |  |
|  | South Mexico City | 25 | 37.3 | 33 | 49.3 | 4 | 6.0 | 3 | 4.5 | 0 | 0 | 1 | 1.5 | 1 | 1.5 | 67 |  |  |  |
|  | East Mexico City | 3 | 6.5 | 23 | 50.0 | 4 | 8.7 | 16 | 34.8 | 0 | 0 | 0 | 0 | 0 | 0 | 46 |  |  |  |
|  | West Mexico City | 28 | 45.9 | 24 | 39.3 | 0 | 0 | 3 | 4.9 | 1 | 1.6 | 2 | 3.3 | 3 | 4.9 | 61 |  |  |  |
|  | State of Mexico | 10 | 17.2 | 34 | 58.6 | 2 | 3.5 | 6 | 10.3 | 4 | 6.9 | 2 | 3.5 | 0 | 0 | 58 |  |  |  |
|  | Other^b^ | 10 | 50 | 5 | 25.0 | 2 | 10.0 | 3 | 15.0 | 0 | 0 | 0 | 0 | 0 | 0 | 20 |  |  |  |
| COVID-19 vaccination scheme | Incomplete | 29 | 31.9 | 39 | 42.9 | 4 | 4.4 | 15 | 16.5 | 1 | 1.1 | 3 | 3.3 | 0 | 0 | 91 | 0.725 |  |  |
|  | Complete | 19 | 30.7 | 25 | 40.3 | 3 | 4.8 | 12 | 19.4 | 1 | 1.6 | 1 | 1.6 | 1 | 1.6 | 62 |  |  |  |
| Comorbidities^c^ | Yes | 28 | 36.4 | 26 | 33.8 | 2 | 2.6 | 17 | 22.1 | 1 | 1.3 | 2 | 2.6 | 1 | 1.3 | 77 | 0.081 |  |  |
|  | No | 49 | 24.4 | 102 | 50.8 | 11 | 5.5 | 28 | 13.9 | 5 | 2.5 | 3 | 1.5 | 3 | 1.5 | 201 |  |  |  |
| Meetings without social distancing | Yes | 33 | 33.0 | 36 | 36.0 | 6 | 6.0 | 19 | 19.0 | 0 | 0 | 5 | 5 | 1 | 1.0 | 100 | **0.003** |  |  |
|  | No | 44 | 24.7 | 92 | 51.7 | 7 | 3.9 | 26 | 14.6 | 6 | 3.4 | 0 | 0 | 3 | 1.7 | 178 |  |  |  |
| Drug Use^d^ | Yes | 7 | 31.8 | 8 | 36.4 | 4 | 18.2 | 3 | 13.6 | 0 | 0 | 0 | 0 | 0 | 0 | 22 | 0.239 |  |  |
|  | No | 69 | 27.1 | 120 | 47.1 | 9 | 3.5 | 42 | 16.5 | 6 | 2.4 | 5 | 2.0 | 4 | 1.6 | 255 |  |  |  |
|  | Unknown/Prefer not to answer | 1 | 100 | 0 | 0 | 0 | 0 | 0 | 0 | 0 | 0 | 0 | 0 | 0 | 0 | 1 |  |  |  |
| Consumption of tobacco-derived products^e^ | Yes | 19 | 25.0 | 37 | 48.7 | 2 | 2.6 | 12 | 15.8 | 4 | 5.3 | 1 | 1.3 | 1 | 1.3 | 76 | 0.447 |  |  |
|  | No | 58 | 28.7 | 91 | 45.1 | 11 | 5.5 | 33 | 16.3 | 2 | 1.0 | 4 | 2.0 | 3 | 1.5 | 202 |  |  |  |
| Diabetes Mellitus | Yes | 5 | 31.3 | 7 | 43.8 | 0 | 0 | 4 | 25.0 | 0 | 0 | 0 | 0 | 0 | 0 | 16 | 0.896 |  |  |
|  | No | 72 | 27.5 | 121 | 46.2 | 13 | 5.0 | 41 | 15.7 | 6 | 2.3 | 5 | 1.9 | 4 | 1.5 | 262 |  |  |  |
| Hypertension | Yes | 11 | 33.3 | 12 | 36.4 | 1 | 3.0 | 8 | 24.2 | 0 | 0 | 0 | 0 | 1 | 3.0 | 33 | 0.537 |  |  |
|  | No | 66 | 26.9 | 116 | 47.4 | 12 | 4.9 | 37 | 15.1 | 6 | 2.5 | 5 | 2.0 | 3 | 1.2 | 245 |  |  |  |
| Obesity | Yes | 5 | 25.0 | 7 | 35.0 | 2 | 10.0 | 4 | 20.0 | 0 | 0 | 2 | 10.0 | 0 | 0 | 20 | 0.155 |  |  |
|  | No | 72 | 27.9 | 121 | 46.9 | 11 | 4.3 | 41 | 15.9 | 6 | 2.3 | 3 | 1.2 | 4 | 1.6 | 258 |  |  |  |
| CD4+ T cell nadir (cells/mm^3^) | < 200 | 32 | 21.8 | 76 | 51.7 | 7 | 4.8 | 27 | 18.4 | 4 | 2.7 | 0 | 0 | 1 | 1 | 147 | **0.019** |  |  |
|  | ≥ 200 | 45 | 34.4 | 52 | 39.7 | 6 | 4.6 | 18 | 13.7 | 2 | 1.5 | 5 | 3.8 | 3 | 2.3 | 131 |  |  |  |
| Previous SARS-CoV-2 infection | Yes | 30 | 24.2 | 62 | 50.0 | 8 | 6.5 | 16 | 12.9 | 6 | 4.8 | 0 | 0 | 2 | 1.6 | 124 | **0.007** |  |  |
|  | No | 47 | 30.7 | 65 | 42.5 | 5 | 3.3 | 29 | 19.0 | 0 | 0 | 5 | 3.3 | 2 | 1.3 | 153 |  |  |  |
|  |  | BNT162b2  (Pfizer/BioNTech) | | AZD1222  (AstraZeneca) | | Ad5-nCoV  (Cansino) | | Gam-COVID-Vac  (Sputnik V) | | CoronaVac  (Sinovac) | | Ad26.COV2-S  (Janssen) | | Spikevax  (Moderna) | |  |  |  |  |
|  |  | **Median** | **(IQR)** | **Median** | **(IQR)** | **Median** | **(IQR)** | **Median** | **(IQR)** | **Median** | **(IQR)** | **Median** | **(IQR)** | **Median** | **(IQR)** |  | **p-value^h^** |  |  |
| CD4+T cell count (cells/mm^3^)^f^ | | 458.5 | (332-592) | 435 | (293-612) | 408.5 | (272-531) | 416.5 | (306-606) | 485 | (255-536) | 461 | (399-618) | 630.5 | (364-890) |  | 0.414 |  |  |
| Interval between doses (days) | | 34 | (30-36) | 60 | (44-63) | 50 | (50-50) | 47 | (32-62) | 37 | (32-45) | NA |  | 33.5 | (30-48) |  | **<0.001** |  |  |
| IQR, interquartile range; ^a^ Row percentages are shown; ^b^ Includes municipalities in other states; ^c^ Includes arterial hypertension, diabetes mellitus, asthma, chronic obstructive pulmonary disease, overweight/obesity, cardiovascular diseases, tuberculosis, liver, kidney and autoimmune diseases; ^d^ Drugs other than alcohol, tobacco and IV drugs; ^e^ Includes cigarette, electronic cigarette and heated tobacco products; ^f^ Results from sample 2; ^g^ Fisher’s exact test, two-sided p-values are shown; ^h^ ANOVA test p-values are shown. | | | | | | | | | | | | | | | | | | |  |
